# Light wavelength-specific regulation of hypersensitive response dynamics in tobacco plants during non-host interaction with *Pseudomonas syringae* pv. *tomato*

**DOI:** 10.1080/15592324.2026.2733228

**Published:** 2026-10-03

**Authors:** Shamjetsabam Gangarani Devi, Nguyen Van Khanh, Yong Hoon Lee

**Affiliations:** a Division of Biotechnology, Jeonbuk National University, Iksan-si, Republic of Korea; b Advanced Institute of Environment and Bioscience, Plant Medical Research Center, and Institute of Bio-industry, Jeonbuk National University, Jeonju-si, Republic of Korea

**Keywords:** Hypersensitive response, green light signaling, reactive oxygen species, non-host resistance, photoregulation of plant immunity

## Abstract

Light quality is a key environmental signal that integrates plant immune pathways, yet how specific wavelengths regulate the execution, timing, and efficiency of the hypersensitive response (HR) remains incompletely resolved. This study investigated how light spectral composition modulates HR dynamics and resistance outcomes during a non-host interaction. Tobacco leaves were inoculated with *Pseudomonas syringae* pv. *tomato* DC3000 (PstDC3000) and incubated under red (RL), green (GL), blue (BL), white light (WL), or darkness. Monochromatic light treatments distinctly reshaped HR progression. BL and WL induced rapid HR characterized by early accumulation of reactive oxygen species (ROS), accelerated electrolyte leakage, and pronounced callose deposition. RL and GL delayed visible HR while effectively restricting bacterial proliferation by moderating ROS production, delaying membrane disruption, and sustaining cell wall reinforcement. In darkness, ROS accumulation was dysregulated, tissue collapse was uncontrolled, and pathogen growth was not effectively constrained. These results demonstrate that light wavelength fine-tunes HR kinetics and qualitatively distinct defense outcomes, with RL and GL favoring controlled immune responses over rapid cell death. By identifying GL as a key modulator of regulated immunity, this study provides a conceptual framework for managing light environments to optimize plant disease resistance.

## Introduction

Plants have evolved sophisticated sensory systems that continuously integrate fluctuating environmental signals with innate immune pathways. Among these signals, light has emerged as a central regulator of plant immunity, extending well beyond its canonical role in photosynthesis. Photoreceptor-mediated signaling intersects with hormone homeostasis, redox regulation, and defense gene expression, establishing light perception as an active determinant of immune outcomes rather than a passive energy source.^1–5^

In incompatible interactions, effector-triggered immunity (ETI) often culminates in the hypersensitive response (HR), a localized and tightly regulated form of programmed cell death that restricts pathogen spread.^6–9^ While HR is a genetically encoded process, its execution is highly plastic. Recent evidence suggests that HR is not a binary “on/off” switch but a highly tunable process in which the timing, magnitude, and spatial constraints of cell death are governed by the environmental context. Several studies have demonstrated that light availability influences HR progression, with darkness frequently associated with runaway cell death and compromised resistance.^10–12^ However, most studies have focused on presence or intensity of light, leaving the role of spectral quality insufficiently resolved.

HR is accompanied by rapid physiological and biochemical changes, including the accumulation of reactive oxygen species (ROS), membrane destabilization, electrolyte leakage, and cell wall fortification via callose deposition. Hydrogen peroxide (H₂O₂) serves as a central signaling molecule that coordinates these responses. Importantly, light not only amplifies ROS production but also governs its spatial and temporal dynamics. Accumulating evidence indicates that individual wavelengths have distinct effects on the plant immune system. Blue light (BL) and red light (RL) have been shown to differentially regulate ROS accumulation, transcriptional reprogramming, and HR progression. In tobacco, RL delays membrane disruption and programmed cell death during non-host interaction with PstDC3000,^2^while in tomato, RL modulates redox-related gene expression during pathogen challenge,^13^ suggesting that light quality may fine-tune HR execution rather than simply enhancing defense intensity. Recently, Tran et al.^5^ reported wavelength-dependent differences in HR progression in tobacco, further supporting the concept that spectral composition influences HR dynamics. Emerging studies now challenge a simplistic view of wavelength effects by demonstrating that green light (GL) modulates immune signaling in a manner distinct from BL and RL.^14^ ^,^ ^15^ GL has been shown to alter disease resistance and defense gene expression despite the lack of a clearly defined GL-specific photoreceptor.^3^ ^,^ ^14^ ^,^ ^15^ However, whether faster HR induced by high-energy BL confers superior resistance compared with more controlled responses under other wavelengths remains unclear.

In this study, HR induced by the non-host pathogen PstDC3000 in tobacco was used as a model to dissect how RL, GL, BL, white light (WL), and darkness differentially configure the execution of ETI. By integrating HR phenotyping with measurements of bacterial growth, ROS accumulation, membrane integrity, callose deposition, and defense-related gene expression, we show that spectral quality governs not only the speed but also the coordination and outcome of HR. The results challenge the prevailing assumption that faster HR is inherently associated with more effective resistance. Instead, we identify wavelength-dependent patterns in which immune signaling is translated into either abrupt tissue collapse or controlled, containment-oriented cell death. In particular, GL emerges as a regulator that biases HR toward a restrained yet effective defense state, highlighting its previously underappreciated role in plant immunity.

## Materials and methods

### Plant material and bacterial growth conditions

Seeds of tobacco (*Nicotiana tabacum* L. cv. Samson) were surface-sterilized with 70% (v/v) ethanol for 1 min, followed by immersion in 1% (w/v) sodium hypochlorite for 15 min, and rinsed five times with sterile distilled water (DW). Sterilized seeds were sown in commercial potting soil in 350 mL plastic pots and grown in a controlled-environment growth room at 28 ± 2 °C under a 16 h light/8 h dark photoperiod. *Pseudomonas syringae* pv. *tomato* DC3000 (PstDC3000) was cultured overnight in Luria Bertani (LB) broth supplemented with rifampicin (50 µg/mL) at 28 °C with continuous shaking at 180  rpm in a shaking incubator under dark conditions. Bacterial cells were harvested by centrifugation (4000 × g, 10 min), washed twice with sterile 10 mM MgCl_2_, and resuspended in 10 mM MgCl_2_ to a final concentration of 1 × 10^7^ CFU/mL.

### Light treatments and HR assay

The effects of light quality on the tobacco–PstDC3000 interaction were evaluated following Nagendran and Lee,^3^ and Rajendran et al.^16^, with minor modifications. Five- to six-week-old tobacco plants were placed in LED growth chambers and pre-acclimated for 3 hours under red light (RL), green light (GL), blue light (BL), or white light (WL) at a photon flux density of 100 µmol m^−2^s^−1^. Peak emission wavelengths were approximately 645 nm (RL), 524 nm (GL), and 458 nm (BL).^3^ ^,^ ^17^ A dark treatment, in which plants were maintained in complete darkness throughout the experiment, was included as a control. Fully expanded leaves were infiltrated on the abaxial surface with bacterial suspension (1 × 10^7^ CFU/mL in 10 mM MgCl_2_) using a needleless syringe. Mock controls were infiltrated with only 10 mM MgCl_2_. Following infiltration, plants were maintained under their respective light conditions at 28 ± 2 °C. Light-treated plants were maintained under a 16 hours light/8 hours dark photoperiod. HR development was visually assessed up to 72 hours after infiltration (hai), and representative images were captured at 6, 12, 24, 48, and 72 hai. The experiment was independently repeated at least three times with similar results.

### Experimental design and tissue sampling

Unless otherwise stated, three independent plants grown in separate pots were used as biological replicates for all tissue-based assays. Two to three fully expanded leaves of similar developmental position on each plant were infiltrated at multiple independent sites to provide sufficient tissue for collection at the designated sampling times. The tissue sampling intervals for each assay were selected according to the temporal characteristics of each HR-associated response, with early time points for rapid biochemical and molecular responses, and extended time courses for HR progression. Assay-specific tissue collection and sample processing are described in the respective sections below.

### In planta bacterial growth assay

Bacterial populations *in planta* were quantified at 6, 12, 24, 48, and 72 hai to monitor bacterial population dynamics throughout HR progression. Two leaf discs (10 mm diameter) were excised from independent infiltration sites of each biological replicate, briefly surface-sterilized with 70% ethanol and rinsed with sterile distilled water. The leaves were pooled and homogenized in 100 µL of 0.45% (w/v) NaCl using a sterile micro-pestle, and serially diluted. Aliquots (100 µL) of appropriate dilutions were plated onto LB agar supplemented with rifampicin (50 µg/mL). Plates were incubated at 28 °C, and colony-forming units (CFU) were counted after 24 hours. Bacterial populations were expressed as Log_10_ CFU/cm^2^ of leaf tissue. Data represent the mean ± SD of three independent biological replicates.

### Electrolyte leakage assay

Electrolyte leakage was measured as described by Hatsugai and Katagiri.^18^ Six leaf discs (10 mm diameter) were collected using a cork borer from independent infiltration sites, at 6, 12, 24, and 48 hai. The leaf discs were gently rinsed in 10 mL sterile ultrapure water for 30 minutes to remove surface electrolytes and then incubated in 10 mL sterile ultrapure water in 50 mL tubes with gentle agitation for 90 minutes at room temperature. The initial electrical conductivity (EC_1_) of the bathing solution was measured using a conductivity meter (HQ14d, Hach). The same leaf discs were subsequently boiled for 10 minutes to release the remaining electrolytes, cooled at room temperature, and the total conductivity (EC_2_) was measured. The electrolyte leakage was calculated as Ion leakage (%) = (EC_1_/EC_2_) × 100. Leaf discs infiltrated with 10 mM MgCl₂ served as mock controls. Data represent the mean ± SD of three independent biological replicates.

### *Histochemical detection and quantification of H*_*2*_ *O*_*2*_

Hydrogen peroxide accumulation was visualized by 3,3′-diaminobenzidine (DAB) staining following Daudi and O’Brien.^19^ Three leaf discs (10 mm diameter) were collected from independent inoculation sites as described above, at 3, 6, and 12 hai and immersed in freshly prepared DAB solution in 12-well plates. These time points were selected to capture early H_2_O_2_ dynamics receding visible HR symptoms and to characterize the initial oxidative burst associated with HR initiation. Vacuum infiltration was performed for 5 minutes, followed by incubation in the dark with gentle shaking (80–100 rpm) for 12 hours. Leaf discs were destained in ethanol: acetic acid: glycerol (3:1:1, v/v/v) at 90–95 °C for 15 minutes, replaced with fresh bleaching solution, and incubated for an additional 30 minutes at room temperature. Samples were mounted on agar plates and photographed against a uniform background.

Quantitative determination of H_2_O_2_ was performed according to Junglee et al.^20^, with minor modifications. Approximately 100 mg of frozen leaf tissue was homogenized in a reaction mixture containing 0.1% (w/v) trichloroacetic acid, 10 mM potassium phosphate buffer (pH 5.8), and 1 M potassium iodide at a 1:1:2 ratio. Control reactions lacking KI were included to correct for background absorbance. Homogenates were centrifuged at 14,000 × g for 15 minutes at 4 °C. Supernatants (200 µL) were transferred to 96-well plates, incubated in the dark at room temperature for 20 minutes, and absorbance was measured at 390 nm. H_2_O_2_ concentrations were calculated using a standard curve and expressed as nanomoles per gram fresh weight (nmol/g FW).

### Callose deposition assay

Callose deposition was analyzed as described by Scalschi et al.^21^, with minor modifications. Three square leaf sections, each encompassing one marked infiltration site, were excised from the infiltrated leaves at 6, 12, and 24 hai. The leaf sections were cleared in 96% ethanol until chlorophyll was completely removed. Samples were rehydrated in 0.07 M sodium phosphate buffer (pH 9.0) for 30 minutes and stained overnight in 0.5% (w/v) methyl blue in the dark. Stained leaves were mounted on microscope slides with 0.05% methyl blue and observed using a fluorescence microscope (Nikon Eclipse Ti2) at 4× magnification with a 475–525 nm excitation filter. Three randomly selected microscopic fields within each leaf section were imaged and analyzed using ImageJ software (version 1.54).^22^ Callose deposition was expressed as the methyl blue-stained area (µm^2^). The mean callose-stained area was calculated for each leaf section from three randomly selected microscopic fields, and the three leaf-section means were subsequently averaged to obtain a single value for each biological replicate.

### Gene expression analysis

Infiltrated leaf tissues pooled from five to six independent infiltration sites of each biological replicate were harvested at 6, 12, and 24 hai, immediately frozen in liquid nitrogen, ground to a fine powder, and stored at −80 °C until RNA extraction. Total RNA was extracted from 75 to 80 mg of ground tissue using the RNAqueous^TM^ Phenol-Free Total RNA Isolation Kit (Invitrogen) according to the manufacturer's instructions. First-strand cDNA was synthesized using the TOPscript^TM^ cDNA Synthesis Kit (Enzynomics^TM^).

Quantitative real-time PCR (qPCR) was performed using a QuantStudio^TM^ 1 Real-Time PCR System (Applied Biosystems) with TOPreal™ SYBR Green qPCR PreMIX (Enzynomics™). Defense- and antioxidant-related genes (*PR1, PR2, Fe-SOD, APX1,* and *GST1*) were amplified using gene-specific primers (Table S1). Cycling conditions consisted of an initial denaturation at 95 °C for 15 minutes, followed by 40 cycles of 95 °C for 15 s, 53 °C for 15 s, and 72 °C for 15 s. Melting-curve analysis was performed to verify primer specificity. Ct values were calculated as the mean of three technical replicates per biological replicate. *β*-Tubulin was used as an internal reference gene. Relative gene expression was calculated using the 2^^−^ΔΔCt^ method,^23^ with mock-treated samples as calibrators. ΔCt values were used for statistical analyzes, while fold-change values are presented graphically.

### Statistical analysis

All experiments were performed with at least three independent biological replicates. Data are presented as mean ± standard deviation (SD). Statistical analyzes were conducted using Excel 2019 with the Real Statistics add-in (Release 8.9.1).^24^ Significant differences among treatments were determined by one-way analysis of variance (ANOVA) followed by Tukey's honestly significant difference (HSD) test at *P* < 0.05.

## Results

### Light wavelength fine-tunes the onset and progression of HR in tobacco leaves

HR is a rapid, localized cell death that restricts pathogen spread during ETI. To determine how light quality modulates HR dynamics, tobacco leaves infiltrated with PstDC3000 were incubated under RL, GL, BL, WL, or darkness. Under dark conditions, water-soaking symptoms appeared as early as 6 hai, indicating rapid tissue collapse without visible lesion containment (Figure 1). In contrast, HR symptoms under BL and WL became visible at 12 hai, whereas RL and GL delayed visible HR until approximately 24 hai. Despite these differences in timing, all light-treated leaves developed characteristic, well-confined necrotic lesions by 72 hai. Dark-treated leaves did not develop clearly defined necrotic lesions and instead showed diffuse tissue damage. These results indicate that light contributes to the coordinated development and spatial containment of visible HR symptoms, while individual wavelengths primarily influence the timing of HR progression rather than the eventual formation of localized lesions.

**Figure 1.**
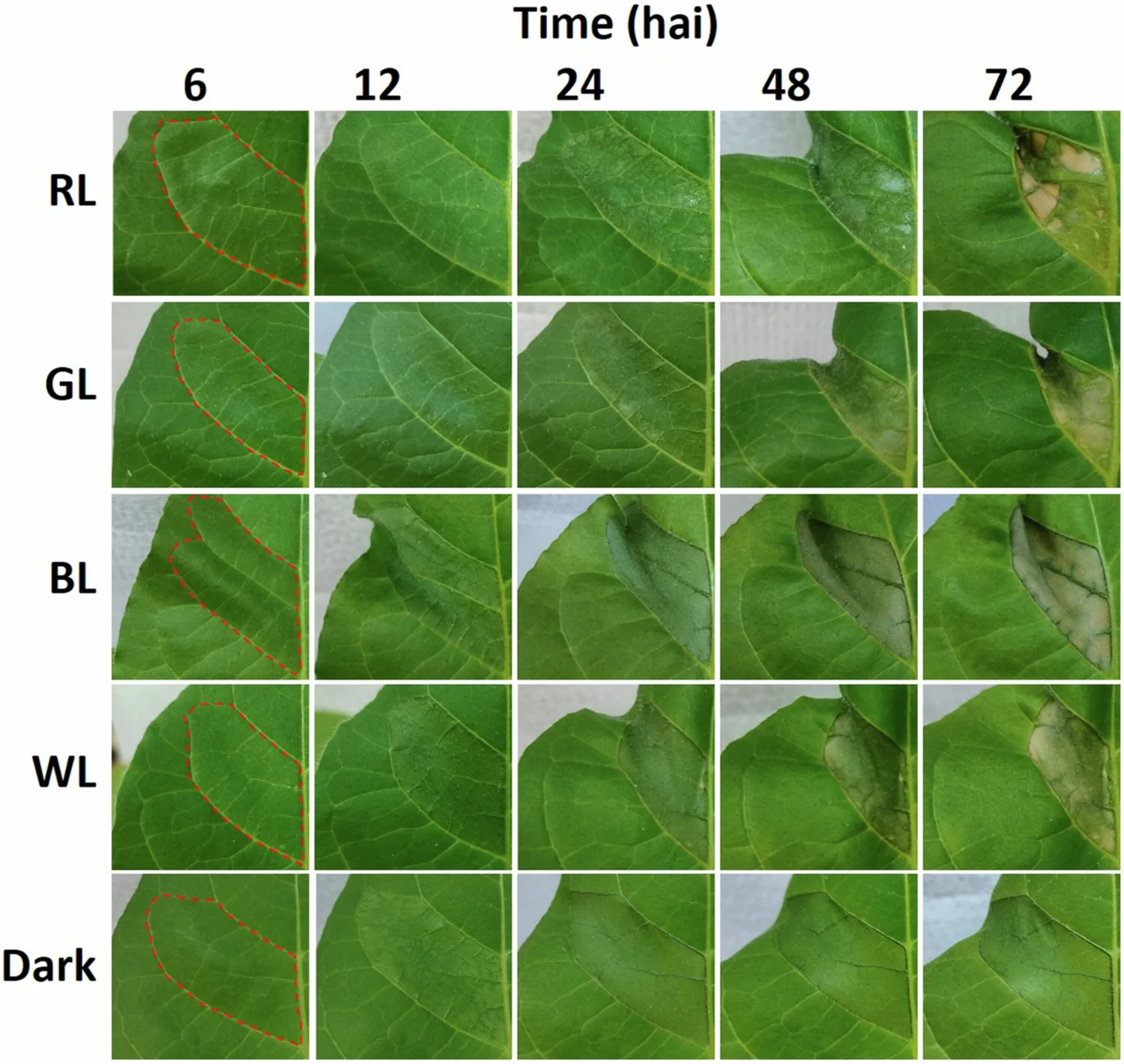
Light wavelength–dependent hypersensitive response in tobacco leaves. Tobacco leaves were infiltrated with *Pseudomonas syringae* pv. *tomato* DC3000 (1 × 10^7^ CFU/mL) and incubated under red (RL), green (GL), blue (BL), white light (WL), or dark conditions. Representative images from three independent experiments are shown at 6, 12, 24, 48, and 72 h after inoculation (hai). The same infiltrated leaf regions were photographed sequentially over time. Red dashed lines at 6 hai indicate the boundaries of the initially infiltrated regions.

### Light quality influences bacterial proliferation during non-host interaction

Because HR is closely associated with the restriction of bacterial growth, *in planta* proliferation of PstDC3000 was quantified under each light treatment. In darkness, bacterial populations increased rapidly to approximately 5.5 Log_10_ CFU/cm^2^ by 12 hai, coinciding with early water-soaking symptoms (Figure 2). Under all light treatments, bacterial populations peaked later, at 24 hai, indicating delayed growth. WL (6.70 Log_10_ CFU/cm^2^) and BL (6.65 Log_10_ CFU/cm^2^) showed the highest bacterial populations at 24 hai, followed by a sharp decline that coincided with rapid HR-associated tissue collapse. In contrast, GL consistently restricted bacterial growth throughout the time-course, resulting in significantly lower populations by 72 hai. RL allowed relatively sustained bacterial populations at later stages. These results indicate that delayed HR under GL does not compromise pathogen restriction, suggesting that HR timing and resistance efficiency can be uncoupled under different light qualities.

**Figure 2.**
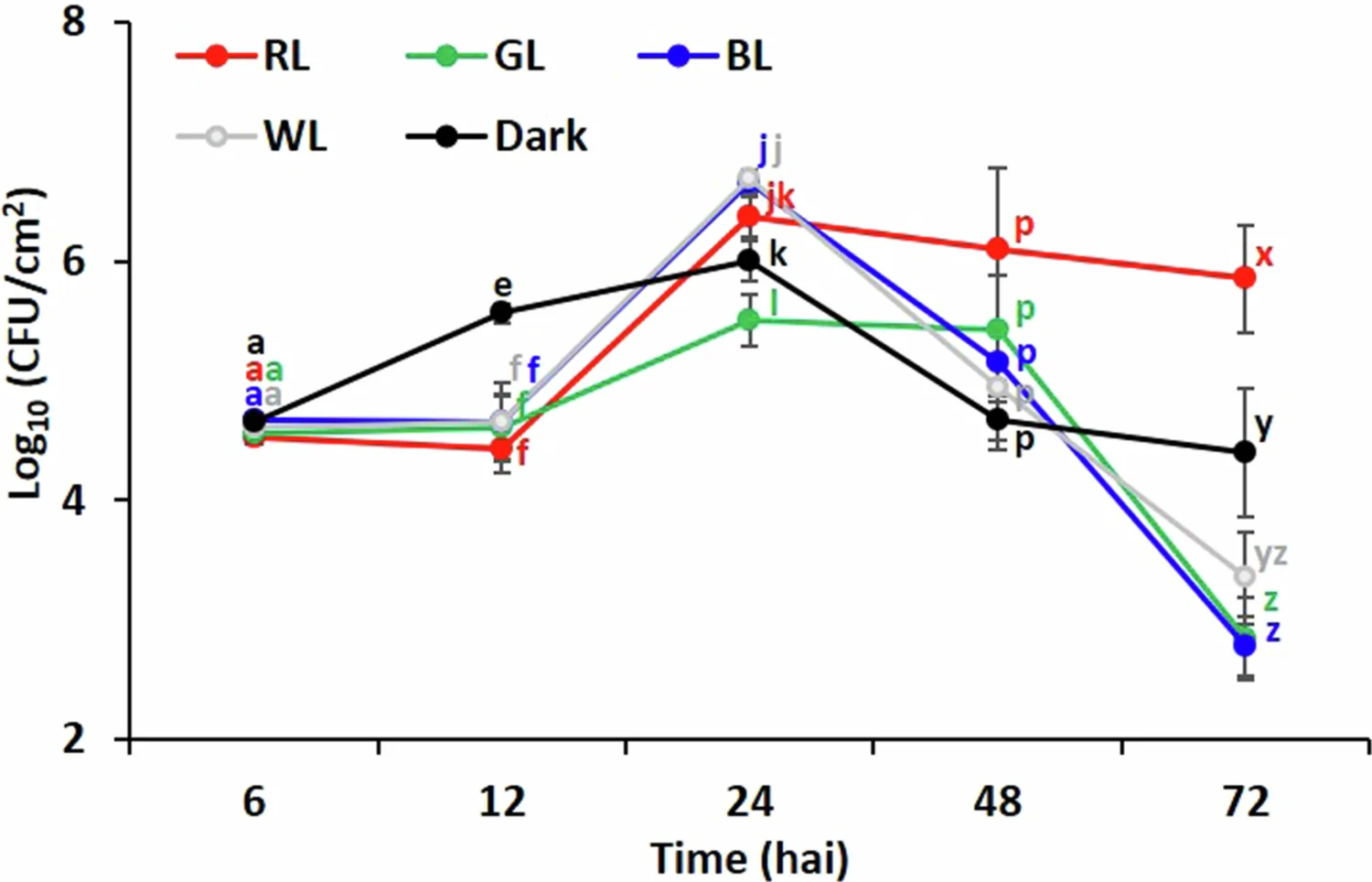
Effect of light wavelength on bacterial growth in tobacco leaves. Bacterial populations of *Pseudomonas syringae* pv. *tomato* DC3000 in infiltrated tobacco leaves incubated under red (RL), green (GL), blue (BL), white light (WL), or dark conditions were quantified by serial dilution plating at 6, 12, 24, 48, and 72 hours after infiltration (hai) and expressed as Log_10_ CFU/cm^2^. Data represent mean ± SD of three independent biological replicates. Different letters indicate significant differences among light treatments at each time point (Tukey's HSD test, *P* < 0.05).

### Light exposure delays membrane disruption during HR-associated cell death

Loss of plasma membrane integrity is a hallmark of HR-associated programmed cell death. To determine whether light quality affects membrane integrity during HR, electrolyte leakage was monitored over time. Dark-treated leaves exhibited the earliest and highest electrolyte leakage, with a sharp increase at 12 hai (Figure 3), indicating rapid and poorly controlled membrane damage. All light treatments delayed electrolyte leakage relative to darkness. WL and BL showed increased leakage at 24 hai, whereas RL and GL reached maximal leakage only at 48 hai. These results indicate that light exposure, irrespective of wavelength, mitigates the rapid onset of HR-associated membrane disruption, with RL and GL in particular promoting a more gradual progression of cell death.

**Figure 3.**
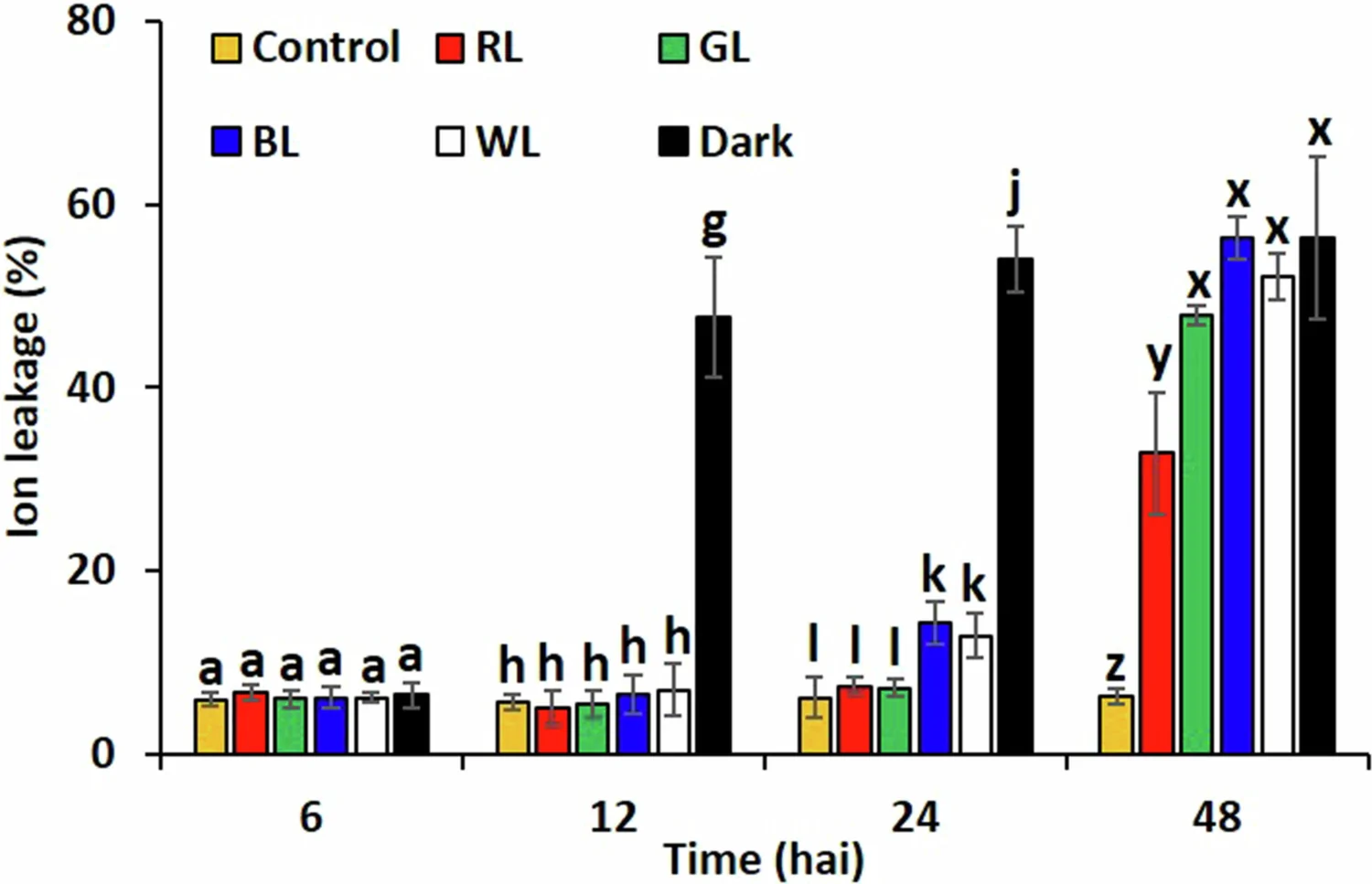
Light-dependent modulation of electrolyte leakage during HR. Electrolyte leakage was measured from leaf discs collected at 6, 12, 24, and 48 hours after infiltration (hai) with *Pseudomonas syringae* pv. *tomato* DC3000 and incubated under different light conditions and expressed as the percentage of total electrolyte leakage. Mock-treated leaves infiltrated with 10 mM MgCl_2_ under WL served as controls. Data represent mean ± SD of three independent biological replicates. Different letters indicate significant differences at each time point (Tukey's HSD test, *P* < 0.05).

### Light wavelength modulates the timing and amplitude of H_2_O_2_ accumulation

ROS, particularly hydrogen peroxide, play central roles in HR signaling and execution. To investigate how light quality affects ROS dynamics, H_2_O_2_ accumulation was analyzed using DAB staining and quantitative assays. Dark-treated leaves showed detectable H_2_O_2_ accumulation as early as 3 hai, followed by BL and WL, which showed early increases (Figure 4; Figure S1). In contrast, RL and GL exhibited delayed ROS accumulation. RL showed a gradual increase, with a marked increase by 12 hai, whereas GL showed an increase in mean H_2_O_2_ accumulation at 6 hai, but became significantly higher than mock control only at 12 hai. Quantitative measurements revealed earlier and stronger H_2_O_2_ accumulation trends under BL and WL, whereas RL and GL showed distinct, delayed temporal profiles of H_2_O_2_ accumulation. These patterns closely mirrored HR kinetics, indicating that light wavelength regulates HR timing by differentially controlling ROS production rather than simply determining its presence.

**Figure 4.**
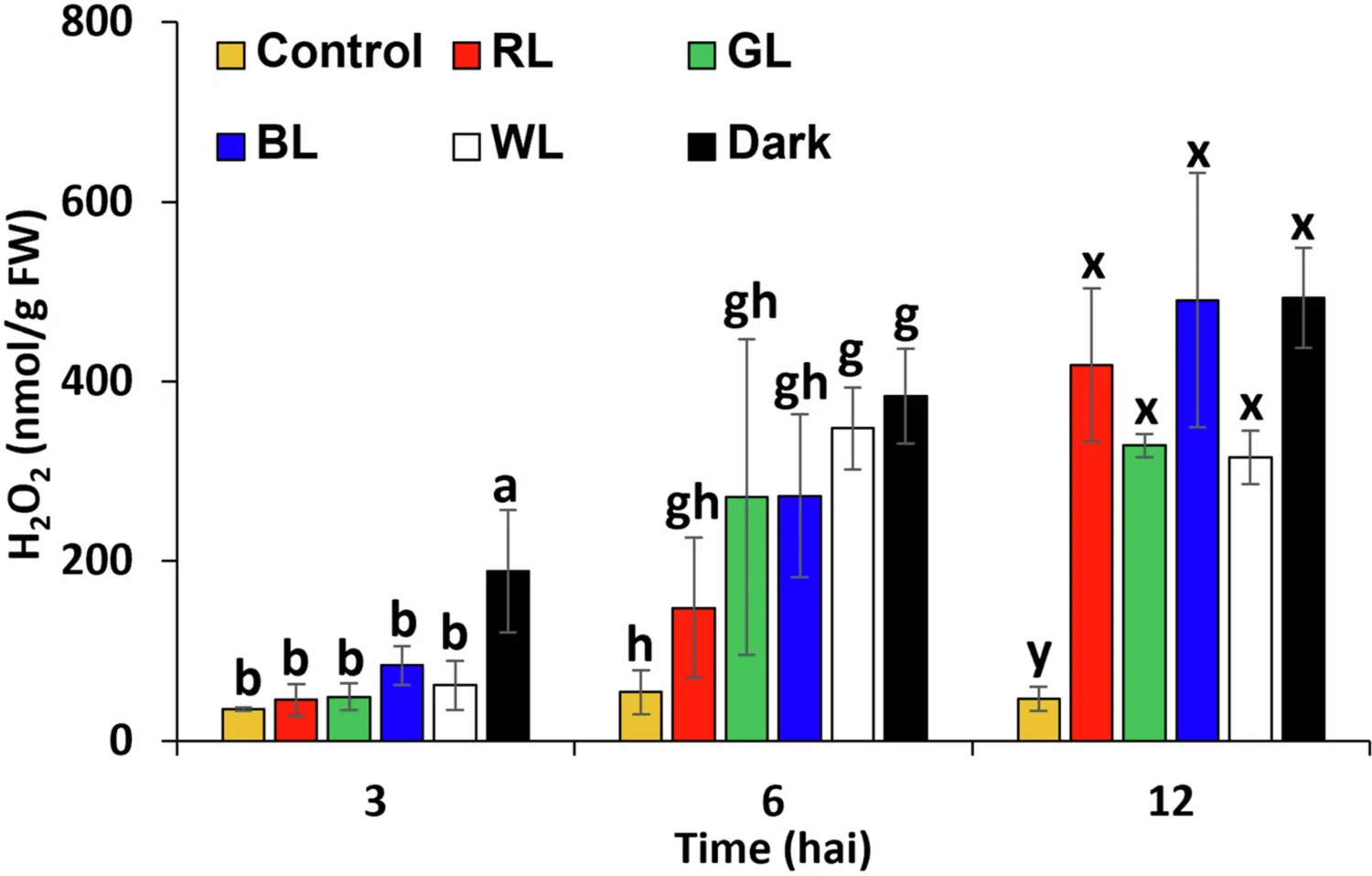
Quantification of hydrogen peroxide (H_2_O_2_) production in pathogen-infected tobacco leaves. Hydrogen peroxide (H₂O₂) levels were quantified in *Pseudomonas syringae* pv. *tomato* DC3000-infected tobacco leaves incubated under red (RL), green (GL), blue (BL), white light (WL), or dark conditions using a potassium iodide -based spectrophotometric assay (absorbance at 390 nm). Mock-treated leaves served as controls. Data represent mean ± SD of three independent biological replicates. Different letters indicate statistically significant differences among treatments at each time point (Tukey's HSD test, *P* < 0.05).

### Light promotes callose deposition during HR

Callose deposition reinforces cell walls and restricts pathogen spread during plant immune responses. To determine whether light quality influences structural defense, callose deposition was quantified. No significant callose deposition was detected at 6 hai under any condition (Figure 5; Figure S2). By 12 and 24 hai, all light-treated leaves showed significantly higher callose deposition than dark-treated leaves. WL induced the highest callose accumulation, followed by BL, GL, and RL. Importantly, despite delayed HR onset, GL-treated leaves exhibited substantial callose deposition at later stages. These results suggest that light-dependent cell wall reinforcement contributes to pathogen restriction even when HR execution is temporally delayed.

**Figure 5.**
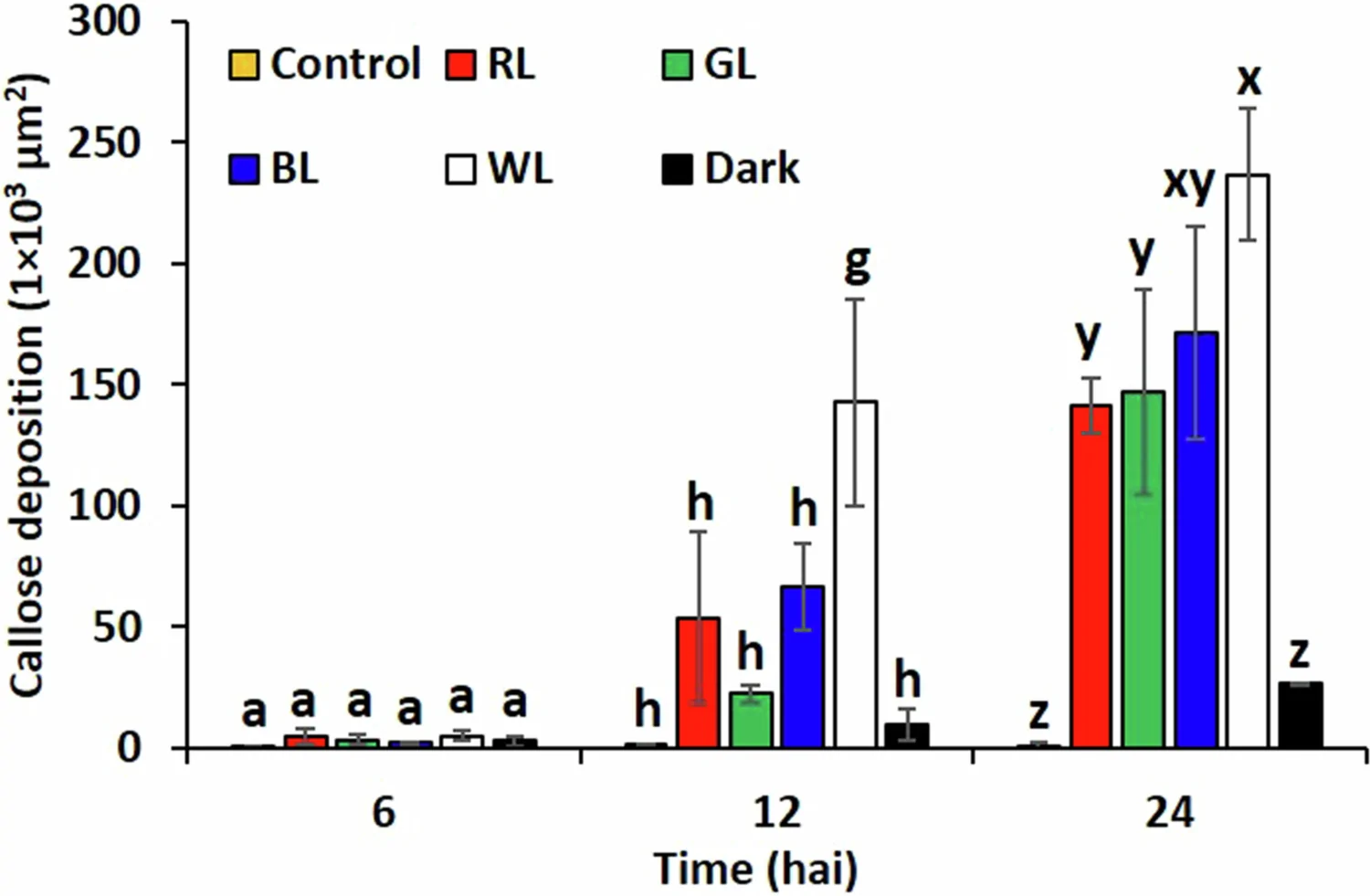
Light-dependent callose deposition in pathogen-infected tobacco leaves. Callose deposition was quantified in tobacco leaves inoculated with *Pseudomonas syringae* pv. *tomato* DC3000 and incubated under red (RL), green (GL), blue (BL), white light (WL), or dark conditions at 6, 12, and 24 hai. Leaves infiltrated with 10 mM MgCl_2_ under WL served as mock controls. Callose accumulation was measured as methyl blue–stained area (µm^2^). Data represent mean ± SD of three independent biological replicates. Different letters indicate significant differences at each time point (Tukey's HSD test, *P* < 0.05).

### Light wavelength differentially regulates defense- and antioxidant-related gene expression patterns

To link physiological responses with transcriptional regulation, defense- and antioxidant-related gene expression was quantified by qPCR. *PR1* and *PR2* were induced under all conditions but displayed wavelength-dependent kinetics (Figure 6A and B). At 12 hai, *PR1* expression was significantly higher under GL than in the mock control, coinciding with controlled HR progression under this wavelength. *PR2* expression was significantly induced under all light treatments, with RL showing the highest mean expression at 12 hai. Dark-treated leaves exhibited delayed but pronounced *PR1* and *PR2* induction at 24 hai.

**Figure 6.**
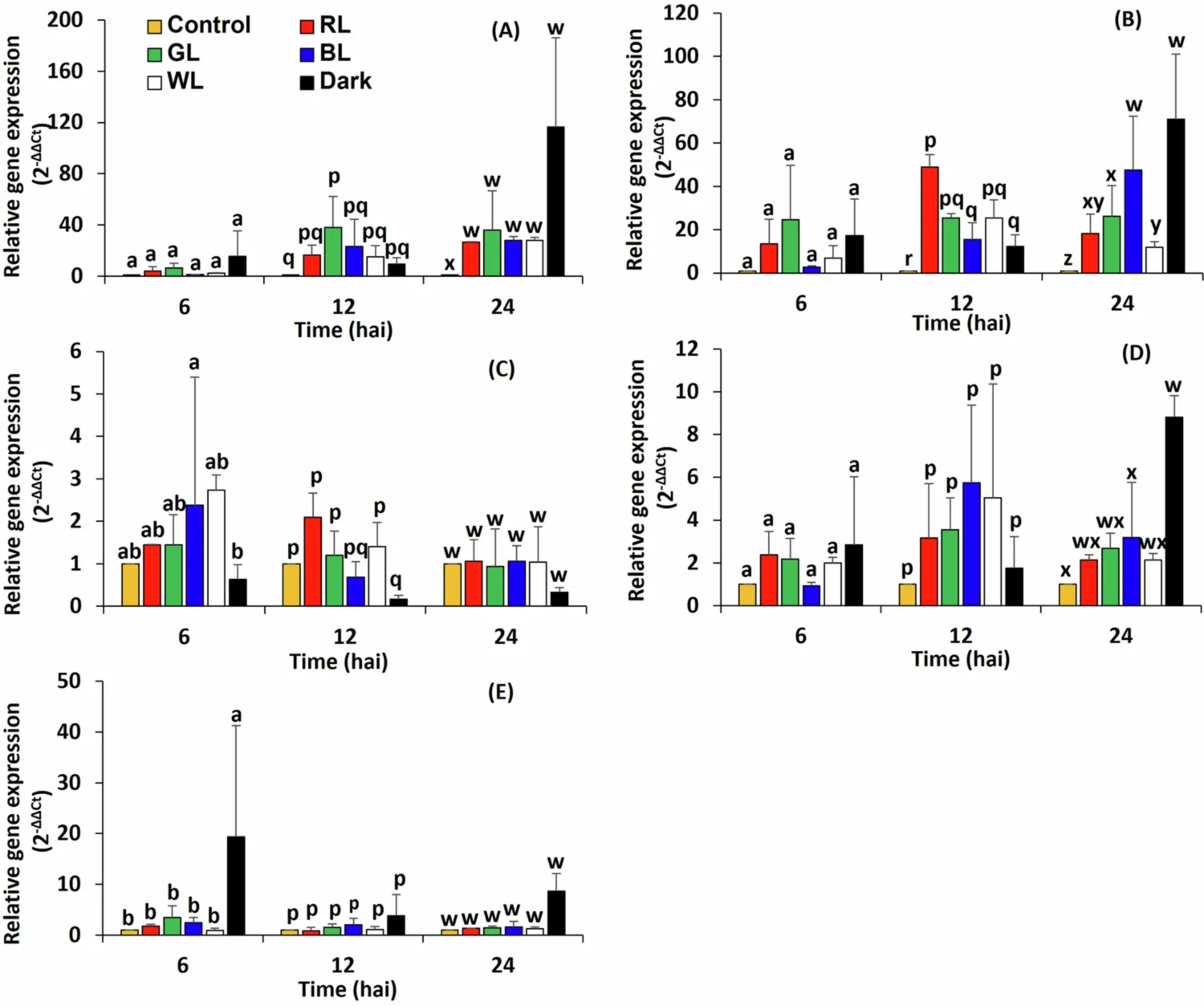
Light wavelength regulates defense- and antioxidant-related gene expression. Relative transcript levels of defense-related genes (A) *PR1* and (B) *PR2*, and antioxidant-related genes (C) *Fe-SOD*, (D) *APX1*, and (E) *GST1* were quantified by qPCR in *Pseudomonas syringae* pv. *tomato* DC3000-infected tobacco leaves incubated under red (RL), green (GL), blue (BL), white light (WL), or dark conditions. Gene expression was normalized to *β*-tubulin and expressed relative to mock-treated controls. Data represent mean ± SD of three independent biological replicates. Different letters indicate significant differences at each time point (Tukey's HSD test, *P* < 0.05).

Antioxidant genes displayed distinct expression profiles (Figure 6C–E). *Fe-SOD* showed higher mean expression at 6 hai under BL and WL, coinciding with early ROS accumulation, but expression returned toward basal levels thereafter and the differences were not statistically significant relative to the mock control. In contrast, *APX1* and *GST1* were strongly induced at 24 hai under darkness, indicating enhanced activation of ROS-scavenging pathways during late HR progression in the absence of light. Collectively, these results indicate that different light wavelengths coordinate defense activation with redox homeostasis, resulting in distinct transcriptional programs associated with HR progression and outcome.

## Discussion

Light is increasingly recognized as an active regulator of plant immune signaling rather than merely an energy source for defense-related metabolism. Genetic and physiological studies in photoreceptor mutants have demonstrated that light perception directly modulates defense signaling networks, including HR-associated programmed cell death.^14^ ^,^ ^25^ ^,^ ^26^ Using HR induced by the non-host pathogen PstDC3000 in tobacco, this study shows that light spectral composition acts as a higher-order regulatory layer that specifies how ETI is executed. Rather than uniformly promoting defense, individual wavelengths reshape the balance among ROS signaling, membrane integrity, cell wall reinforcement, and defense gene activation. Our findings therefore suggest that light quality regulates not only the timing of HR but also the coordination between cell death and pathogen restriction.

BL and WL promoted rapid HR-associated responses, whereas RL and GL delayed HR development while still supporting localized lesion formation and pathogen restriction. Darkness, in contrast, promoted early water-soaking and membrane disruption but resulted in less clearly defined HR-associated tissue damage. These results extend previous light-versus-dark studies^10^ ^,^ ^12^ by demonstrating that spectral composition modulates HR execution, ranging from a well-coordinated immune response to uncontrolled tissue collapse.

Tran et al.^5^ also reported that different light wavelengths modulate HR progression in tobacco, emphasizing differences in HR execution speed and oxidative damage. In their study, GL- and BL-treated plants exhibited reduced programmed cell death, lower H₂O₂ accumulation, and substantially less membrane damage compared with plants under WL. However, in our experimental system, BL promoted relatively rapid HR progression, whereas GL consistently delayed HR development. Despite this distinction, both studies collectively demonstrate that spectral composition plays a fundamental role in regulating HR execution and plant defense responses.

Our results demonstrate that faster HR is not necessarily associated with greater resistance. Although BL and WL accelerated HR onset, they were also associated with rapid membrane disruption and relatively high bacterial populations during early HR progression. RL—and particularly GL—delayed HR execution while maintaining effective bacterial restriction. This uncoupling of HR timing from resistance outcome advances previous wavelength-based analyzes by showing that the speed of HR is a poor proxy for resistance. Instead, the data support a model in which RL and GL promote a more regulated immune response that balances ROS signaling, structural defenses, and host tissue integrity. In this context, GL emerges as a condition that supports defense execution while minimizing collateral damage, rather than a weak or passive wavelength.

ROS, particularly H_2_O_2_, are central regulators of HR, acting as both local executioners and signaling molecules.^27–29^ Consistent with these roles, BL and WL induced strong and early H₂O₂ accumulation, closely correlating with accelerated HR lesion formation and rapid electrolyte leakage. These effects align with the activation of chloroplast- and apoplastic ROS-generating systems by cryptochromes and phototropins.^30–32^ However, despite higher early mean *Fe-SOD* expression under BL and WL, early ROS bursts were accompanied by rapid membrane destabilization. This suggests that aggressive oxidative signaling may compromise tissue integrity and thereby disrupt sustained execution of the defense response. This may partly explain the relatively high bacterial population observed during early HR progression under these wavelengths. In contrast, RL and GL delayed ROS accumulation without abolishing defense activation. This delayed ROS profile is consistent with a distinct redox signaling regime, potentially involving phytochrome-mediated modulation of defense thresholds and salicylic acid–dependent pathways.^26^ ^,^ ^33^ Traditionally, GL has been considered biologically weak; however, recent studies and the present data indicate that GL is a potential modulator of HR timing and immune signaling, despite the absence of a dedicated GL-specific photoreceptor.^3^ ^,^ ^14^ ^,^ ^15^ The effectiveness of pathogen restriction under GL indicates that moderate and temporally controlled ROS signaling may be more advantageous than rapid oxidative bursts, supporting reports that excessive ROS can exacerbate host damage without proportionally enhancing resistance.^11^ ^,^ ^34^

Electrolyte leakage analyzes further revealed that light wavelength regulates not only HR onset but also the precision of programmed cell death execution. BL and WL induced rapid and extensive membrane disruption, whereas RL and GL delayed leakage, indicative of restrained and coordinated cell death. Darkness produced the earliest and strongest membrane leakage yet failed to develop clear necrotic lesions, resembling runaway cell death phenotypes.^35^ These results indicate that light, particularly RL and GL, supports the regulated execution of HR, with membrane destabilization occurring in coordination with defense signaling rather than as a consequence of nonspecific damage. Such precise coordination is critical for effective HR and has been reported in other light-dependent immune responses.^12^

Callose deposition followed a clear wavelength-dependent pattern, being strongest under WL, followed by BL, GL and RL, and minimal in darkness. This pattern broadly corresponded with ROS dynamics and HR progression, consistent with the roles of ROS and salicylic acid in callose synthesis.^36–38^ Despite delayed HR onset, GL-supported callose deposition coincided with effective bacterial containment, indicating that structural defenses may compensate for slower execution of cell death. Defense- and antioxidant-related gene expression further underscored wavelength-specific immune strategies. BL and WL showed higher early mean *Fe-SOD* expression, consistent with rapid HR initiation and oxidative stress. In contrast, *PR1* expression peaked earliest and most strongly under GL, whereas *PR2* showed prominent response under RL, suggesting that these wavelengths promote timely activation of defense genes. Together with callose deposition, the observed transcriptional profiles under GL and RL point to a more balanced defense strategy that restricts pathogen growth while limiting excessive oxidative damage.

Overall, our results support a model in which light perception modulates HR by differentially regulating ROS dynamics, membrane integrity, structural defenses, and defense gene expression. BL and WL favor rapid HR execution driven by early ROS accumulation and strong callose deposition, whereas RL and particularly GL are associated with delayed yet effective HR, characterized by distinct ROS dynamics and preserved tissue integrity. Darkness disrupts this coordination, resulting in poorly regulated cell death and compromised resistance (Figure 7). Together, these findings demonstrate that HR speed alone is not a reliable indicator of immune effectiveness. Instead, the wavelength of light determines how precisely HR is executed and how effectively resistance is achieved. By revealing GL as a signal that balances the trade-off between pathogen restriction and collateral host damage, this work reframes green light from a marginal to a regulatory component of plant immune signaling. Future studies should identify the photoreceptors and downstream modules that interpret RL and GL and define how these pathways intersect with canonical ROS and hormonal networks. Such studies could advance an integrated view of light-guided immune regulation in crop protection.

**Figure 7.**
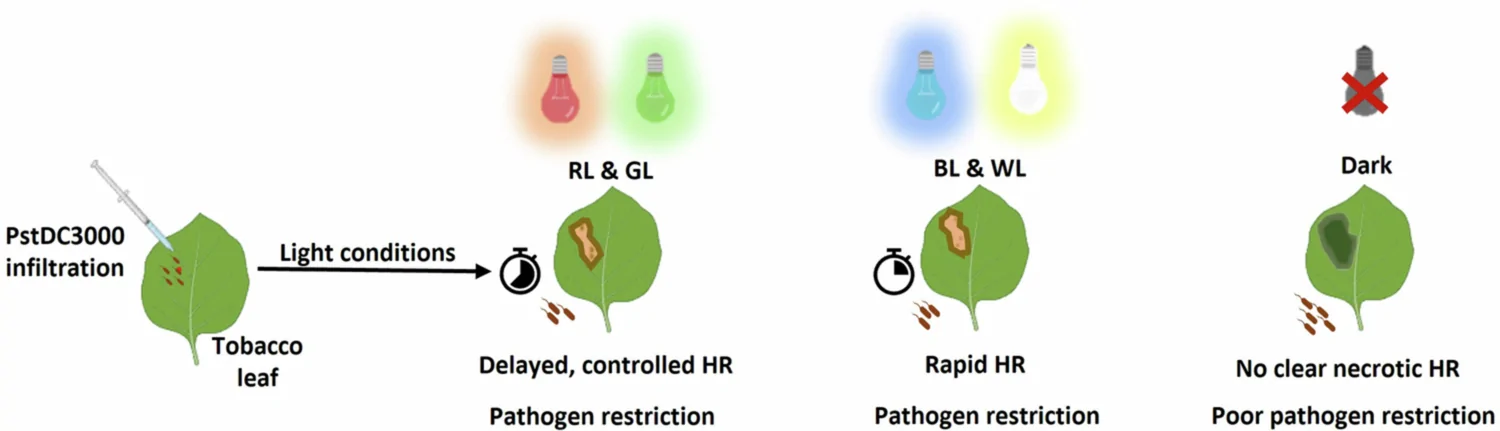
Proposed schematic model of wavelength-dependent regulation of the hypersensitive response in tobacco during non-host interaction with *Pseudomonas syringae* pv. tomato DC3000. The model summarizes how spectral quality differentially modulates the timing and coordination of HR-associated immune responses. RL and GL are associated with delayed but controlled HR, whereas BL and WL promote rapid HR; both response patterns result in pathogen restriction. In darkness, the absence of light-mediated signaling is associated with uncoordinated defense responses, the absence of clear necrotic lesions, and poor pathogen restriction.

## Supplementary Material

Supplementary Material 3_Supplementary_Data.FINAL.docx

## Data Availability

The data that support the findings of this study are available on request from the corresponding author.
