# Supplementary material for: Light wavelength-specific regulation of hypersensitive response dynamics in tobacco plants during non-host interaction with Pseudomonas syringae pv. tomato
Source: Plant Signal Behav. 2026 Oct 3;21(1):2733228. doi: 10.1080/15592324.2026.2733228 (PMC13644593; doi:10.1080/15592324.2026.2733228)
Supplement: Supplementary file 1 [file KPSB_A_2733228_SM2924.docx]

**Supplementary data**

Fig. S1. Histochemical detection of hydrogen peroxide accumulation. Tobacco leaves were infected with *Pseudomonas syringae* pv. *tomato* DC3000 and incubated under different light wavelengths: red light (RL), green light (GL), blue light (BL), white light (WL) or dark conditions. Leaf disks (10 mm diameter) were excised at 3, 6, and 12 h after incubation (hai) and stained with DAB to visualize H_2_O_2_ accumulation. Leaves infiltrated with 10 mM MgCl_2_ served as negative controls. Representative images from three independent experiments are shown.

Fig. S2. Light-dependent callose deposition in pathogen-infected tobacco leaves. Five-six-week-old tobacco leaves were inoculated with *Pseudomonas syringae* pv. *tomato* DC3000 and incubated under red light (RL), green light (GL), blue light (BL), white light (WL), or dark conditions. Leaves infiltrated with 10mM MgCl_2_ served as mock control. Representative fluorescence micrographs of callose accumulation from three independent experiments are shown. Scale bar = 300 µm.

Table S1. Primers for used for this study

| Name | Primers (5’→3’) | Source |
| --- | --- | --- |
| *PR1* | F: TAGCTGAGGGAAGTGGCGAT  R: TCCACACACCTGTCCTTGTG | Tran and Jung (2020) |
| *PR2* | F: TTAGCGAATACCAACCCGCC  R: ACATTGGCTAAGAGTGGAAGGT | Tran and Jung (2020) |
| *Fe-SOD* | F: CACACCGCTCCTCACCATAG  R: GACTGCTTCCCATGACACGA | Tran and Jung (2020) |
| *APX1* | F: TTTGAGCGGGGAAAAGGAGG  R: CTCAACCAATGGGCGGAAGA | This study |
| *GST1* | F: GGAGGAAAGGGTTGGTCCTG  R: AAGTGGGAAACTCGGACACC | This study |
| *β-Tubulin* | F: GCATCTTTGCGTACACTTTGC  R: ACAACTCGAAACCAACGCTT | Tran and Jung (2020) |
